# Notes and News

**Published:** 1893-11-04

**Authors:** 


### NOTES AND NEWS.

COLLECTED AND COMMUNICATED.

THE number of hospitals in this country has shown a considerable increase lately. At Nottingham Lady Belper opened a hospital for women. The premises are not new, having been previously used as Inland Revenue offices. They have undergone complete alteration, and are arranged to accommodate from twenty-five to thirty beds. The building and site cost £3,500, and the alterations and furnishing £5,000 more. The whole of this sum has not been provided, and therefore the income of the hospital will be rather heavily taxed. At the opening ceremony a collection of £641, in purses and otherwise, was made.

AN eye infirmary for North Durham was opened by the Marchioness of Londonderry. The building is situated with free spaces on either side, and a garden at the rear. Much of the furnishing has been especially provided by local friends of the institution. Accommodation is made for sixteen patients. The whole cost of the out-patients' department, amounting to £1,200 has been subscribed by the working men of the town. The opening ceremony was an interesting one, celebrated by several entertainments in aid of the institution. At Plymouth the new premises of the Homœopathic Hospital were opened by Lord Morley. The outlay is estimated at £2,000, towards which large sums have already been subscribed.

ALL institutions benefitting by the demonstrations of trade and friendly societies are naturally too grateful for the funds they get, to speculate upon what they might get, were these parades managed economically. However, the question has been raised by a correspondent in the *Echo*, who is very dissatisfied with the result of the collections for the miners' wives and children. He points out that the public have no idea how much goes in display on these occasions. In another part of the same paper figures are given showing that on one particular occasion a collection of £97 only produced £54 for the two hospitals benefited.

THE Glasgow Sanitary Authorities have directed that ice-cream manufacturers should register themselves as vendors of milk, it being found that infection can be conveyed by congealed milk. One tradesman has already been prosecuted for non-compliance with the rule.

THE strange outbreak of illness so closely resembling cholera in its symptoms is now comparatively over at the Greenwich Workhouse. Although Dr. Klein cannot trace any certain indications of cholera, every precaution has been taken to disinfect as thoroughly as if the epidemic had been of a virulent nature.

AT a further meeting of the Camberwell Committee of the Hospital Saturday Fund, fresh evidence as to the discrepancies existing between the amounts known to have been in the boxes of the collectors and on the receipts given, was brought forward. Counsel's opinion why no prosecution should be commenced was read, a new Secretary was appointed in place of Mr. Allen, and a vote of thanks to Mr. Hobbs for his interest in the fund was passed.

IT seems to us a very great waste of the funds of institutions to advertise annual meetings in the *Times* and other important journals, when the management treat such meetings as private, to the exclusion of reporters, who desire to attend with the object of benefiting the institution. A reporter, attending the annual meeting of the Magdalen Hospital, Streatham, which was publicly advertised, was refused admission, and informed that it was not customary to admit reporters to the meetings of this hospital. We should be glad to have from the management some explanation of this unusual proceeding.

AT a special court at the Radcliffe Infirmary, Oxford, the resignation of the Treasurer, Dr. Bright, was formally read. Dr. Bright has held office for ten years, during which time he has shown great activity and interest on behalf of the institution, of which the meeting expressed itself duly appreciative. At the same court a new treasurer, Lord Dillon, was appointed. The appointment is a happy one, for Lord Dillon is popular both in the county and the city, and he has had touch with hospital work previously through his connection with an important metropolitan hospital. Further, his speech on accepting office speaks hopefully for his influence at the Radcliffe. He acknowledges the necessity of being in the "front rank" and of educating the public to give.

WE are glad to note that the result of the inquiry into the complaints against the Smallpox Hospital, West Ham, confirmed our view that the objections existed in the past rather than the present. Reading between the lines, it is easy to conclude that no one will vouch for the perfection of the hospital of to-day, but in view of the improvements that have already been effected, and the strong light which has been brought to bear on the institution, we feel that it is but right that the hospital should now be given an opportunity to gain the confidence of the neighbourhood. It has been suggested to us that in our previous note the distinction between the smallpox hospital and the West Ham General Hospital was not sufficiently clearly drawn, we feel assured, however, that the smallpox hospital complaints have been made too public, and the merits of the general hospital are too well known for any mistake in our meaning to have been made.

